# Ten-year trends of hypertension treatment and control rate in Korea

**DOI:** 10.1038/s41598-021-86199-x

**Published:** 2021-03-26

**Authors:** Kwang-il Kim, Eunjeong Ji, Jung-yeon Choi, Sun-wook Kim, Soyeon Ahn, Cheol-Ho Kim

**Affiliations:** 1 Department of Internal Medicine, Seoul National University College of Medicine, Seoul, Republic of Korea; 2 Department of Internal Medicine, Seoul National University Bundang Hospital, Gumi-ro 173-82, Bundang-gu, Seongnam, Kyeongi 463-707 Republic of Korea; 3 Medical Research Collaborating Center, Seoul National University Bundang Hospital, Seongnam, Republic of Korea

**Keywords:** Hypertension, Epidemiology

## Abstract

We analyzed the Korean National Health and Nutrition Examination Survey (KNHANES) database to determine the trends of hypertension treatment and control rate in Korea over the past 10 years. In addition, we tried to investigate the effect of chronic medical conditions on hypertension management. We investigated the hypertension prevalence, awareness, treatment, and control rate from 2008 to 2017. KNHANES, which uses a stratified multistage sampling design, is a cross-sectional, nationally representative survey conducted by the Korean government. A total of 59,282 adults (≥ 20 years) were included, which was representative of the total population of around 40 million Koreans per year. The mean age was 50.7 ± 16.4 years and 42.6% were male. The prevalence of hypertension, hypercholesterolemia, diabetes mellitus, and obesity significantly increased over the 10 years. During this period, the hypertension treatment and control rate significantly improved. Hypertension treatment rate was significantly lower in the younger age group compared to the older age group, but the control rate among the treated patients was not significantly different between age groups. The treatment and control rates of hypertension were higher in patients with multimorbidity, which implies that it has a favorable effect on the treatment and control of hypertension. Hypertension treatment and control rate have improved over the past 10 years. The higher treatment and control rate in patients with multimorbidity suggest that the more aggressive surveillance might be associated with the improvement of hypertension treatment and control rate in Korea.

## Introduction

Hypertension is a major risk factor for cardiovascular disease. Furthermore, blood pressure ≥ 120/80 mmHg leads to a greater risk of myocardial infarction, heart failure, stroke, and kidney disease. Although many nations tried to decrease the burden of hypertension, the number of individuals with elevated systolic blood pressure (≥ 110–115 mmHg) and the estimated associated deaths have increased substantially worldwide^1^. A recent paper about the hypertension control rate in 12 high-income countries reported that hypertension awareness, treatment, and control rate have substantially improved since the 1980s and 1990s, most of which was achieved in the late 1990s and early 2000s. However, control rates have plateaued in the past decade, at levels lower than those seen in high-quality hypertension control programs^2^.

It has been reported that the prevalence of hypertension has remained stable over time. In the US, the prevalence of hypertension was unchanged from 1999 to 2016. The prevalence of controlled hypertension increased from 1999 to 2010 but then showed no change until 2016^3^. This is disappointing since during the previous 10-year period, the percentage of uncontrolled hypertension among adults who were taking prescribed hypertension medication decreased by 8%^4^.

In Korea, hypertension awareness, treatment, and control rate dramatically improved from 1998 to 2007, but have remained almost unchanged thereafter^5^. The awareness rate increased from 25% in 1998 to 65% in 2007, but remained at 65% in 2016. The treatment rate increased from 22% in 1998 to 59% in 2007 and 61% in 2016, whereas the control rate increased from 5% in 1998 to 41% in 2007 and to 44% in 2016.

All these data suggest that it is urgent to overcome the recent stagnation in hypertension control and to find a novel strategy to decrease the global burden of elevated blood pressure. To do that, it is important to identify factors associated with poor hypertension control. For this purpose, we investigated the trend of hypertension awareness, treatment, and control rate in the Korean population. We also tried to identify factors associated with hypertension management. More specifically, we looked into the effect of multimorbidity on the treatment and control rate of patients with hypertension in Korea.

## Methods

### Study population

The data were derived from the Korean National Health and Nutrition Examination Survey (KNHANES). The KNHANES has been conducted periodically since 1998 to assess the health and nutritional status of non-institutionalized Koran civilians. The KNHANES is a cross-sectional and nationally representative survey conducted by the Division of Chronic Disease Surveillance, Korea Disease Control and Prevention Agency (KDCA). The survey consists of a health interview, nutrition, and health examination surveys. A stratified, multistage probability sampling design was used for the selection of household units. More specific design features and characteristics of KNHANES are described elsewhere in detail^6^.

Among the 64,853 subjects older than 20 years who participated in the survey between 2008 and 2017, 5571 subjects were excluded due to lack of data regarding blood pressure or hypertension treatment. Finally, 59,282 subjects, including 25,240 males (42.6%) were included in the analysis. All study procedures were carried out in accordance with relevant guidelines and regulations. All the surveys are conducted with the participants’ written consent. This study was approved by the Institutional Research Committee of Seoul National University Bundang Hospital (IRB No. X-1908-559-903).

### Measurement

Blood pressure was measured by a trained nurse using a mercury sphygmomanometer (Baumanometer Desk model 0320, W. A. BAUM, Copiague, NY) with an appropriately sized cuff after the participants sat for at least 5 min to stabilize blood pressure. During the measurement period, participants were seated leaning against the back of a chair with the feet flat on the floor. The participants’ right arm was positioned so that the middle of the cuff was at the heart level. Blood pressure was measured 3 times and the mean blood pressure of second, third measured value was used in the analysis of systolic and diastolic blood pressure.

A fasting blood sample was taken in the morning after at least 8 h of fasting. The blood samples were centrifuged, refrigerated at the examination site, and transferred in iceboxes to a central laboratory. Blood chemistry tests were performed by appropriate methods using an auto-analyzer (Hitachi Automatic Analyzer 7600, HITACHI, Tokyo, Japan) (Supplementary Table S1). The body mass index (BMI) was calculated as the weight in kilograms divided by the height in meters squared.

### Definitions

Hypertension was defined as blood pressure ≥ 140/90 mmHg or current treatment with antihypertensive drugs. Hypertension awareness was defined by having a medical diagnosis of hypertension by medical personnel. Hypertension treatment was defined if the participants were taking antihypertensive medications for ≥ 20 days/month. Hypertension control was defined as having an average systolic and diastolic blood pressure of < 140/90 mmHg. Control rate was defined as the proportion of participants with adequate blood pressure control among those with hypertension or taking antihypertension medication.

Hypercholesterolemia was defined as total cholesterol ≥ 240 mg/dl or current treatment with cholesterol lowering drugs. Diabetes mellitus was defined as fasting glucose ≥ 126 mg/dl or treatment with oral agents or insulin^7^. Obesity was defined as BMI ≥ 25 kg/m^2^.^8^

The GFR (glomerular filtration rate) was calculated using the chronic kidney disease-epidemiology collaboration (CKD-EPI) equation and impaired GFR was defined as an eGFR of 15–59 ml/min/1.73m^2^)^9^.

### Statistical analysis

Statistical analyses were done with SPSS (version 15.0, SPSS Inc., Chicago, IL) or SAS (version 9.4, SAS Institute, Cary, NC). To calculate the total population that the sample would represent, we used stratified variables and sample weights designed by the KDCA. In order to confirm the changes in prevalence, we combined every two-year data using the integrated weight. Continuous variables were expressed as mean ± standard error or median and interquartile range and compared by unpaired Student’s t-test and one-way ANOVA. Discrete variables were expressed as counts and percentages, and the Rao-Scott χ^2^ was used to compare proportions. Uni- and multivariable logistic models were used to identify factors affecting prevalence and hypertension treatment. The temporal trends of the prevalence of chronic medical conditions as well as hypertension treatment and control rate were examined using the slope of the regression line. All statistical analyses were two-tailed and p-values < 0.05 were considered statistically significant.

## Results

### Basic characteristics of participants

**Figure 1:**
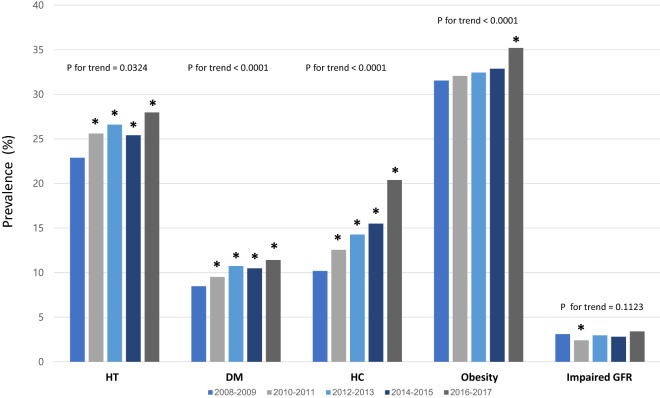
Trends of hypertension, diabetes mellitus, hypercholesterolemia, obesity, and impaired GFR prevalence from 2008 to 2017 in Korea. Prevalence of hypertension, diabetes mellitus, hypercholesterolemia, and obesity is significantly increased from 2008 to 2017. *HT* hypertension, *DM* diabetes mellitus, *HC* hypercholesterolemia, *GFR* glomerular filtration rate, *p < 0.05 compared with 2008–2009. Trend analysis was performed by regression analysis and age-adjusted P values are presented.

A total of 59,282 adults (≥ 20 years) were included in the analysis. The characteristics of study population are listed in Table 1. The age-adjusted prevalence of hypertension increased over the 10-year study period (Fig. 1). The hypertension prevalence was 23.0 ± 0.51% in 2008–2009 but increased to 27.7 ± 0.58% in 2016–2017. The age-adjusted prevalence of other chronic medical conditions such as diabetes mellitus (8.5–11.6%), hypercholesterolemia (10.0–20.6%), and obesity (31.6–35.2%) increased during the period as well. However, there was no significant change in age-adjusted prevalence of impaired GFR (3.1–3.3%).

**Table 1:** Clinical and laboratory characteristics of KNHANES participants.

	2008–2009 (N = 14,123)	2010–2011 (N = 12,116)	2012–2013 (N = 10,783)	2014–2015 (N = 10,094)	2016–2017 (N = 12,166)	P value
Age	44.92 (0.27)	45.60 (0.32)	46.08 (0.30)	46.82 (0.30)	47.48 (0.29)	< .0001
Male sex	5993 (49.47%)	5184 (49.30%)	4489 (49.00%)	4259 (48.77%)	5315 (49.65%)	0.6562
Hypertension	3853 (23.02%)	3695 (25.31%)	3419 (26.23%)	3228 (25.65%)	3969 (27.71%)	< .0001
Diabetes mellitus	1415 (8.48%)	1354 (9.37%)	1403 (10.71%)	1330 (10.30%)	1700 (11.57%)	< .0001
Hypercholesterolemia	1539 (10.00%)	1711 (12.33%)	1653 (13.98%)	1784 (15.71%)	2713 (20.56%)	< .0001
Obesity	4462 (31.59%)	3840 (31.96%)	3461 (32.58%)	3327 (32.68%)	4269 (35.25%)	< .0001
Impaired GFR	589 (3.07%)	402 (2.37%)	427 (2.84%)	409 (2.74%)	549 (3.31%)	0.0025
Current smoker	3127 (27.08%)	2523 (26.67%)	2047 (24.21%)	1780 (22.18%)	2188 (21.83%)	< .0001
Waist circumference (cm)	81.08 (0.15)	81.16 (0.16)	80.77 (0.17)	81.79 (0.15)	82.38 (0.15)	< .0001
BMI(kg/m^2^)	23.62 (0.04)	23.66 (0.05)	23.76 (0.05)	23.78 (0.05)	23.96 (0.05)	< .0001
SBP (mmHg)	115.44 (0.27)	117.29 (0.27)	117.23 (0.28)	116.82 (0.26)	117.78 (0.23)	< .0001
DBP (mmHg)	75.30 (0.17)	75.35 (0.17)	75.53 (0.18)	74.95 (0.17)	75.85 (0.14)	< .0001
Fasting glucose (mg/dL)	97.02 (0.25)	96.43 (0.27)	97.69 (0.29)	98.80 (0.30)	99.79 (0.30)	< .0001
Cholesterol (mg/dL)	186.27 (0.46)	188.02 (0.50)	188.41 (0.50)	188.91 (0.48)	193.52 (0.45)	< .0001
TG (mg/dL)	135.65 (1.29)	134.39 (1.45)	135.70 (1.46)	137.82 (1.47)	140.40 (1.68)	< .0001
BUN (mg/dL)	14.28 (0.06)	13.86 (0.07)	14.01 (0.06)	14.16 (0.06)	14.16 (0.06)	< .0001
Creatinine (mg/dL)	0.86 (0.004)	0.84 (0.002)	0.86 (0.004)	0.84 (0.004)	0.84 (0.003)	< .0001
AST (IU/L)	22.24 (0.15)	22.31 (0.15)	22.15 (0.19)	22.58 (0.15)	22.76 (0.16)	0.0002
ALT (IU/L)	22.49 (0.21)	21.89 (0.20)	22.58 (0.35)	22.09 (0.24)	22.70 (0.21)	0.0007

Data are presented as number (weighted %) or weighted mean (SE).

*GFR* glomerular filtration rate, *BMI* body mass index, *SBP* systolic blood pressure, *DBP* diastolic blood pressure, *TG* triglyceride, *BUN* blood urea nitrogen, *AST* aspartate aminotransferase, *ALT* alanine aminotransferase.

P values are calculated by one-way ANOVA for continuous variables and by Rao-Scott χ^2^ for discrete variables.

### Changes in hypertension awareness, treatment, and control rate

**Figure 2:**
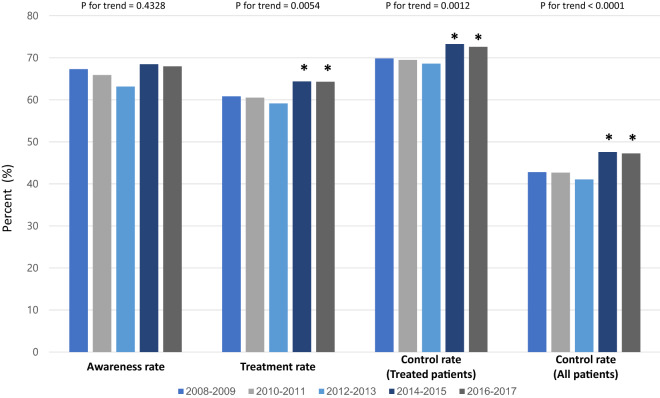
Trends of hypertension awareness, treatment, and control rate from 2008 to 2017. Hypertension treatment and control rate have been significantly improved from 2008–2009 to 2014–2017. *p < 0.05 compared with 2008–2009. Trend analysis was performed by regression analysis and age-adjusted P values are presented.

**Figure 3:**
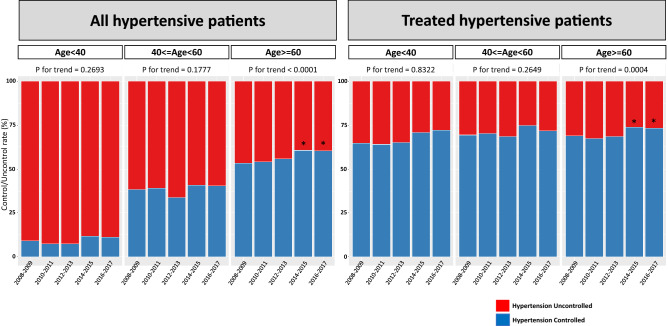
Hypertension control rate according to age group. The hypertension control rate is higher in middle age (40–60 years) and older age (≥ 60 years) group compared with younger age (< 40 years) group. However, among the treated patients, there was no significant difference according to age group. Hypertension control rate has been improved among the older age (≥ 60 years) group; however, there was no significant improvement in other age group. *p < 0.05 compared with 2008–2009. Trend analysis was performed by regression analysis and age-adjusted P values are presented.

Hypertension treatment and control rate improved over the last 10 years, but there was no significant improvement in the age-adjusted hypertension awareness rate. Hypertension control rate was 41.6 ± 1.24% in 2008–2009 and increased to 47.3 ± 1.17% in 2016–2017 for all hypertensive patients. In addition, the hypertension control rate among treated patients improved from 69.3 ± 1.26% in 2008–2009 to 73.0 ± 1.12% in 2016–2017 (Fig. 2). Hypertension awareness and treatment rate showed a greater improvement among male patients. Unfortunately, the awareness, treatment, and control rate of hypertension in the younger age group (20–39 years old) and middle age group (40–59 years old) did not change during the 10-year period; in contrast, the hypertension control rate significantly improved in the older age group (≥ 60 years) (Fig. 3).

### Comparison between non-treated and treated patients with hypertension

There were significant differences in accompanying co-morbidities and laboratory results between non-treated and treated patients with hypertension. Compared with the non-treated hypertensive patients, treated hypertensive patients were older and predominantly male. Other chronic medical conditions such as diabetes mellitus, hypercholesterolemia, obesity, and impaired GFR were more common among the treated patients. Interestingly, the blood cholesterol and triglyceride levels were significantly lower in the treated hypertensive patients, which suggest that those patients might be treated with lipid-lowering therapy as well. To identify the independent factors associated with hypertension treatment, we performed multiple logistic regression analysis, Younger age, male sex, and current smoker were associated with hypertension non-treatment. In contrast, presence of diabetes mellitus and hypercholesterolemia, and increased waist circumference were related with hypertension treatment (Table 2).

**Table 2:** Uni- and multivariable logistic regression analysis to identify factors affecting hypertension treatment.

	**Univariable analysis**				**Multivariable analysis**			
	95% CI				95% CI			
	OR	lower	upper	P value	OR	lower	upper	P value
Age	0.925	0.922	0.929	< .0001	0.934	0.93	0.938	< .0001
Male sex	2.229	2.065	2.407	< .0001	1.628	1.461	1.814	< .0001
Diabetes mellitus	0.302	0.272	0.334	< .0001	0.521	0.445	0.611	< .0001
Hypercholesterolemia	0.417	0.378	0.459	< .0001	0.364	0.323	0.411	< .0001
Obesity	0.928	0.861	1	0.0501				
Impaired GFR	0.202	0.168	0.242	< .0001	0.811	0.655	1.003	0.0529
Current smoker	2.434	2.21	2.681	< .0001	1.271	1.126	1.434	0.0001
Waist circumference (cm)	0.988	0.983	0.992	< .0001	0.97	0.964	0.975	< .0001
BMI (kg/m^2^)	0.994	0.982	1.005	0.2652				
Glucose (mg/dL)	0.99	0.988	0.992	< .0001	1.001	0.999	1.004	0.2888
Cholesterol (mg/dL)	1.01	1.009	1.012	< .0001	1.015	1.014	1.017	< .0001
TG (mg/dL)	1.001	1.001	1.002	< .0001	1	0.999	1	0.0468
BUN (mg/dL)	0.913	0.899	0.927	< .0001	0.959	0.943	0.975	< .0001
Creatinine (mg/dL)	0.948	0.844	1.066	0.3734				
AST (IU/L)	1.006	1.002	1.01	0.0011	1.004	0.998	1.009	0.2202
ALT (IU/L)	1.013	1.01	1.015	< .0001	1	0.996	1.004	0.945

*OR* odds ratio, *CI* confidence interval, *GFR* glomerular filtration rate, *SBP* systolic blood pressure, *DBP* diastolic blood pressure, *BMI* body mass index, *WBC* white blood cell, *TG* triglyceride, *BUN* blood urea nitrogen, *AST* aspartate aminotransferase, *ALT* alanine aminotransferase.

### Effect of multimorbidity on hypertension treatment and control rate

**Figure 4:**
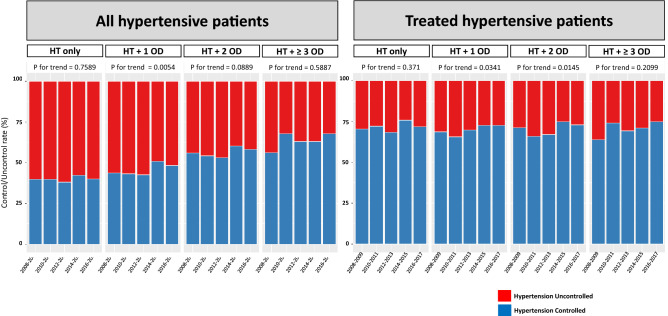
Hypertension control rate according to number of accompanying chronic medical conditions. The hypertension control rate is higher in patients with other medical condition than hypertension only patients. Number of co-morbidities represents the sum of diabetes mellitus, hypercholesterolemia, obesity, and impaired GFR (range; 0–4). *HT* hypertension, *OD* other disease. Trend analysis was performed by regression analysis and age-adjusted P values are presented.

**Figure 5:**
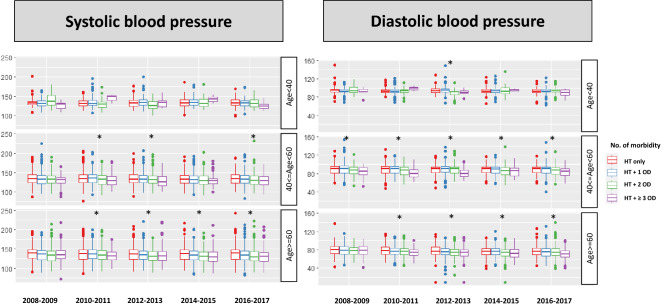
Trends of systolic and diastolic blood pressure in patients with hypertension according to accompanying other medical conditions. Systolic and diastolic blood pressures are inversely correlated with the number of chronic medical conditions among the middle age (40–60 years) and older age (> = 60 years) group. *OD* other disease, *p < 0.05 by ANOVA.

Among the patients with hypertension, 29.6% had only hypertension; however, the remaining 70.4% also had at least one other chronic medical condition. The prevalence of multimorbidity increased in recent years. Among patients with hypertension, 65.4% had more than one other chronic disease in the 2008–2009 period, which increased to 74.1% in 2016–2017. Additionally, the pattern of multimorbidity was different according to the age of participants; more participants had multiple morbidity with increasing age (Supplementary Fig. S1). Having other chronic disease had a significant effect on the treatment and control rate of hypertension. Compared to patients with only hypertension, patients who had other chronic medical conditions such as diabetes mellitus, hypercholesterolemia, obesity, and impaired GFR were more likely to be treated and controlled for hypertension. Moreover, the age-adjusted control rate was improved in patients with hypertension and one or two other chronic medical conditions. In contrast, the hypertension control rate among treated patients was not different according to the presence of chronic medical condition, but the control rate also significantly improved in recent years (Fig. 4). The systolic and diastolic blood pressure of patients with hypertension decreased during the past 10 years, which was more prominent among the older group (age ≥ 60 years) and in patients with other chronic medical conditions (Fig. 5).

## Discussion

We showed that (1) the prevalence of hypertension and other chronic medical conditions increased during the last 10 years; (2) compared with the middle or older age groups, the hypertension control rate was lower in the younger age group, mainly due to the lower treatment rate; (3) the hypertension control rate significantly improved in hypertensive patients having other medical conditions, suggesting that multimorbidity has a beneficial effect on hypertension treatment or control.

Since the KNHANES participants were representative of the Korean population, we can conclude that the awareness, treatment, and control rate of Korean patients with hypertension improved during the past 10 years. To overcome the recent stagnation in hypertension treatment and control rate, we should focus on the younger age group and on patients without other medical conditions to improve the awareness and treatment rate.

As a result of national projects for the improvement of hypertension control, there has been a substantial advance in the treatment of hypertension throughout the world. Among US adults, there was a decrease in age-adjusted hypertension prevalence, although the absolute burden increased. The age-adjusted control rate improved from 1999 to 2016 among adults treated for hypertension. However, the control rate did not improve among older adults or in patients with hypertension and diabetes mellitus, CKD, or high cardiovascular disease risk^10^.

In our study, the systolic and diastolic blood pressure levels were significantly lower in the older population, especially in patients with multiple comorbidities. Recent guidelines highlighted the importance of strict blood pressure control for patients with high cardiovascular risk or established cardiovascular disease. In addition, many guidelines recommended a target blood pressure of 130/80 mmHg instead of 140/90 mmHg for those patients^11–13^. Accordingly, there has been a substantial decline in systolic and diastolic blood pressure in patients with hypertension and a high-risk profile. There was a rapid increase in the proportion of patients whose blood pressure met the criterion of less than 140/90 mmHg, even in octogenarians, in a study of primary care electronic health records of 265,225 participants from the UK Clinical Practice Research Datalink. That study showed that hypertension treatment intensified between 2001 and 2014. Blood pressure values decreased in both treated and untreated participants, with a substantial increase in the proportion of patients achieving conventional blood pressure targets among octogenarians^14^.

Hypertension rarely manifests alone; it is usually clusters with other chronic medical conditions, such as diabetes mellitus, obesity, hypercholesterolemia, and CKD. This clustering independently increases the risk of developing cardiovascular disease, even in individuals with high-normal blood pressure^15^. As a result, patients with hypertension and other cardiovascular risk factors are regarded as a high-risk group and show resistance to hypertension treatment^16^.

In Korea, the clinical profile of patients with hypertension has been worsening over the last 10 years, namely in terms of the increased prevalence of comorbidities such as diabetes, obesity, and hypercholesterolemia. Moreover, the blood pressure, blood glucose, cholesterol, and BMI also significantly increased during this period. These data support the finding that the overall cardiovascular risk is currently higher compared to previous years in the Korean population.

However, the awareness, treatment, and control rate of hypertension have improved, especially in older patients with multiple medical conditions. These findings have been observed in other studies as well. In the PRESCAP study, the clinical profile of treated hypertensive patients has progressively worsened over the past decade in Spanish primary care settings. Despite that, blood pressure control rates significantly improved during that period^17^. An improvement in hypertension control was also observed in Sweden^18^. More specifically, blood pressure decreased more among patients with greater cardiovascular risk. This constitutes a contradictory finding because previous data suggested that hypertensive patients having multimorbidity show resistance to drug treatment. This may be due to increased physicians’ awareness and intensification of hypertension treatment for high-risk patients. The importance of intensive blood pressure control for these patients or those with other chronic medical conditions has been highlighted in most of the clinical guidelines. Recent treatment guidelines are now beginning to incorporate the concept of global cardiovascular risk evaluation and management to improve patient outcomes and to recommend global risk assessment in all patients with hypertension^12,13^. Accordingly, the improvement in hypertension treatment and decrease in blood pressure might be associated with the change of primary care physicians’ attitude.

On the contrary, in this study, lower awareness and treatment rate were seen in the younger age group (20–39 years old) consistently during the past 10 years. However, the control rate among the treated patients was comparable to that of older age group. This result suggests that the low awareness and treatment rate in the younger age group should be overcome. In Korea, blood pressure higher than 130/80 mmHg among young adults has been associated with adverse clinical outcomes^19^. In addition, low-risk patients with stage I hypertension also showed unfavorable cardiovascular outcomes, but hypertension control had a beneficial effect even in low-risk Korean patients.^20^ Previously, many physicians focused on the treatment of high-risk older patients. However, considering the poor treatment rate among younger patients, a new nationwide program should be implemented to enhance the hypertension awareness and treatment rate targeting younger patients. In line with this perspective, May Measurement Month (MMM), which is a global awareness campaign initiated by the International Society of Hypertension (ISH), put the spotlight on increasing access to blood pressure screening as potentially the most effective way to reduce hypertension’s adverse toll on health^21^. In addition, smartwatch or other wearable device have potential to detect hypertension early, thus possibly to improve the awareness of hypertension especially among the younger population.^22^ Further research whether smartwatch or wearable device based self-measurement of blood pressure can improve the awareness or treatment of hypertension should be developed and conducted.

This study has limitation. The definition of hypertension treatment was taking antihypertensive medications for ≥ 20 days/month; however, the data was obtained by the questionnaire. More accurate methods such as prescription check are required to assess the compliance of antihypertensive medication.

In conclusion, hypertension treatment and control rate improved during the past 10 years in Korea. The higher hypertension treatment and control rate in patients with comorbidity suggest that more aggressive surveillance or treatment for these patients might be associated with the improvement in hypertension treatment and control rate in Korea. Further efforts to increase the treatment rate for younger patients without other medical conditions should be provided for the better control of hypertension in Korea.

## Supplementary information

Supplementary information.
