# Supplementary material for: Ten-year trends of hypertension treatment and control rate in Korea
Source: Sci Rep. 2021 Mar 26;11:6966. doi: 10.1038/s41598-021-86199-x (PMC7997987; doi:10.1038/s41598-021-86199-x)
Supplement: Supplementary file 1 — Supplementary information. [file 41598_2021_86199_MOESM1_ESM.pdf]

Supplementary table 1. Detailed information regarding blood chemistry tests used in KNHANES

	Method	Analyzer
Cholesterol	Enzymatic method	Hitachi Automatic Analyzer 7600 (Hitachi/Japan)
Glucose	Hexokinase UV	Hitachi Automatic Analyzer 7600 (Hitachi/Japan)
TG	Enzymatic method	Hitachi Automatic Analyzer 7600 (Hitachi/Japan)
BUN	Kinetic UV assay	Hitachi Automatic Analyzer 7600 (Hitachi/Japan)
Creatinine	Kinetic colorimetric assay	Hitachi Automatic Analyzer 7600 (Hitachi/Japan)
AST	UV method	Hitachi Automatic Analyzer 7600 (Hitachi/Japan)
ALT	UV method	Hitachi Automatic Analyzer 7600 (Hitachi/Japan)

TG, triglyceride; BUN, blood urea nitrogen; AST, aspartate aminotransferase; ALT, alanine aminotransferase

Supplementary Figure 1. Pattern of multimorbidity according to age group and year

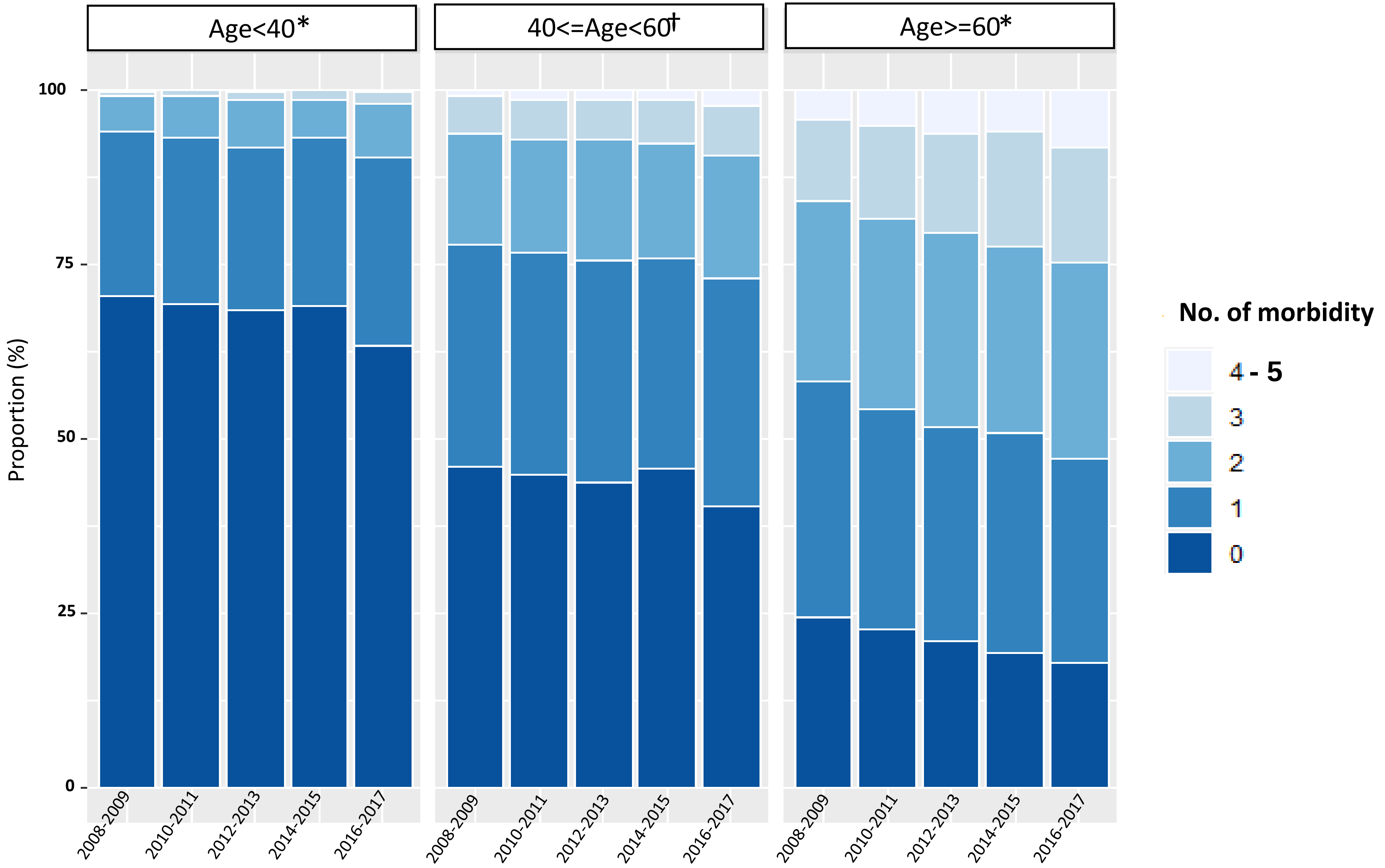

Multimorbidity is prevalent as people get older, which is more prominent in recent years. *;P < 0.0001, †; P=0.0002 by Rao-Scott Chi-Square Test.
